# Desulfurization of diesel *via* joint adsorption and extraction using a porous liquid derived from ZIF-8 and a phosphonium-type ionic liquid†

**DOI:** 10.1039/d3re00364g

**Published:** 2023-08-04

**Authors:** Chenhua Shu, Min Zhao, Hua Cheng, Yajie Deng, Pierre Stiernet, Niklas Hedin, Jiayin Yuan

**Affiliations:** a School of Chemistry and Environmental Science, Shangrao Normal University Shangrao 334001 China; b Department of Materials and Environmental Chemistry, Stockholm University Stockholm 10691 Sweden chenhua.shu@mmk.su.se jiayin.yuan@mmk.su.se

## Abstract

A type-III porous liquid based on zeolitic imidazolate framework-8 (ZIF-8) and an ionic liquid trihexyltetradecylphosphonium bis(trifluoromethylsulfonyl)imide ([THTDP][BTI]) was synthesized and used for the desulfurization of model diesel. The desulfurization effect by ZIF-8/[THTDP][BTI] combined both the adsorptive desulfurization by ZIF-8 and the extraction desulfurization by [THTDP][BTI]. The removal of the three chosen aromatic organic sulfides by the ZIF-8/[THTDP][BTI] porous liquid followed the order of dibenzothiophene (73.1%) > benzothiophene (70.0%) > thiophene (61.5%). It was further found that deep desulfurization could be realized by ZIF-8/[THTDP][BTI] through triple desulfurization cycles and ZIF-8/[THTDP][BTI] can be regenerated readily. The desulfurization mechanism was explored further in detail by conformation search and density functional theory calculations. Calculations supported that the large molecular volume of [THTDP][BTI] excluded itself from the cavities of ZIF-8, making the pores of ZIF-8 in the porous liquid unoccupied and accessible by other guest species, here the studied organic sulfides. These calculations indicate that the van der Waals interactions were the main interactions between ZIF-8/[THTDP][BTI] and specifically benzothiophene. This work supports that the porous liquid ZIF-8/[THTDP][BTI] could potentially be used for desulfurization of diesel in industry.

## Abstract 2

A type-III porous liquid was prepared through dispersing ZIF-8 in the ionic liquid [THTDP][BTI] and was used for the desulfurization of model diesel.

## Graphical abstract

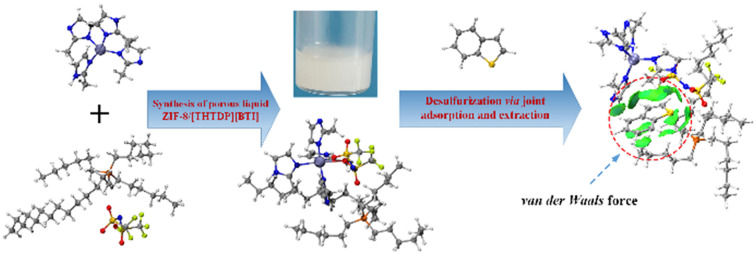

## 1. Introduction

Organic sulfides in fuel oil poison automotive three-way catalysts, leading to increased emissions of hydrocarbons, CO and NO_*x*_. Moreover, the emission of SO_2_ increases, which increases the risk of acid rain, smog, *etc.* Therefore, stringent control of the sulfur content of fuel oil is in place in most countries.^1^ Currently, hydrodesulfurization (HDS) is the default desulfurization technology in the refining industry. However, it requires a high temperature and pressure and the consumption of hydrogen is high. HDS has significant issues about the removal of aromatic organic sulfides such as thiophene (Th), benzothiophene (BT), dibenzothiophene (DBT), and their derivatives.^2^ Furthermore, HDS can saturate the olefins in fuel oil, thereby decreasing the octane number of fuel oil.^3^ In this regard, non-HDS methods such as adsorptive desulfurization,^4^ extractive desulfurization,^5^ oxidative desulfurization,^6^ and biological desulfurization^7^ have been the focus of current research. Among them, adsorptive desulfurization is regarded as one of the most promising desulfurization methods for fuel oil due to its simple hydrogen-free and oxidant-free process, ease of operation and low investment and operational costs.^8^ The adsorbents used for the desulfurization of fuel oil are generally solid sorbent materials including activated carbons, metal oxides, and metal–organic frameworks (MOFs).^9–11^ However, solid sorbent materials lack flowability and are incompatible with industrial pump systems designed to operate liquids. In addition, solid sorbents suffer from several disadvantages, *e.g.*, physical aging, plasticization and mechanical fatigue.^12^

Porous liquids, an emerging class of materials first proposed by James and co-workers in 2007, can circumvent the aforementioned disadvantages of solid sorbent materials.^13^ Porous liquids refer to a group of liquids with permanent porosity, which combines the permanent pore structure of solid sorbent materials with the continuous flowability of liquids.^14–16^ Due to their excellent attributes, porous liquids have been increasingly studied and used in separation, catalysis, electrolysis, and so on.^17–19^ In particular, a large amount of research activities on the application of porous liquids have been dedicated to studies of gas separation/capture.^20–25^ There have also been several studies on fuel oil desulfurization using porous liquids. For example, Wu *et al.* constructed a series of porous liquids using zeolitic imidazolate framework-8 (ZIF-8) and pyridinium- and imidazolium-type ionic liquids for fuel oil desulfurization.^26^ Yu *et al.* used MIL-53(Al) and dicationic imidazolium-based ionic liquids to synthesize porous liquids for fuel oil desulfurization.^27^ Zhang *et al.* reported porous liquids derived from quaternary ammonium salt functionalized silica for fuel oil desulfurization.^28^ Despite the advances achieved in these previous studies, there remain challenges with porous liquids for fuel oil desulfurization. For example, some ionic liquids for synthesizing porous liquids such as imidazolium-based ionic liquids are costly and chemically unstable due to the C-2 proton, for long-term and large-scale industrial use.^29^ In addition, some synthetic processes of porous liquids in these studies, *e.g.*, functionalized silica-based porous liquids, are too complicated to be industrialized for fuel oil desulfurization.

This work aims to address the above-mentioned problems in previous studies and in turn promote the application of porous liquids in fuel oil desulfurization. A type-III porous liquid, termed “ZIF-8/[THTDP][BTI]”, was adopted and studied for the desulfurization of model diesel in this work. The less expensive and chemically robust phosphonium-based ionic liquid trihexyltetradecylphosphonium bis(trifluoromethylsulfonyl)imide ([THTDP][BTI]) was chosen for synthesizing a porous liquid,^30,31^ as it has been proven as an effective, sterically hindered solvent for synthesizing type-III porous liquids due to its large molecular size;^32^ in addition, generally phosphonium-based ionic liquids are regarded as promising solvents for sulfur extraction from liquid fuels.^33–35^ It can be expected that ZIF-8/[THTDP][BTI] could benefit from each component for efficient desulfurization, *i.e.*, the adsorptive desulfurization by the solid component ZIF-8 and the extractive desulfurization by the liquid component [THTDP][BTI]. Furthermore, the porous liquid ZIF-8/[THTDP][BTI] can be synthesized simply through ultrasonic dispersion of ZIF-8 in [THTDP][BTI]. Therefore, the porous liquids derived from phosphonium-type ionic liquids and ZIF-8 can serve as promising candidates for future industrial desulfurization use.

## 2. Experimental

### 2.1 Materials and methods

Zinc acetate dihydrate (≥99%), 2-methylimidazole (98%), methanol (≥99.5%) and *n*-tetradecane (99%) were purchased from Adamas Reagent Co. Ltd. [THTDP][BTI] (99%) was purchased from Ningbo Yuanli New Materials Co. Ltd. Th (97%), BT (97%) and DBT (98%) were purchased from Aladdin Reagent Co. Ltd., China.

### 2.2 Synthesis of ZIF-8 and porous liquid

ZIF-8 was synthesized according to a previous report.^24^ Briefly, zinc acetate dihydrate and 2-methylimidazole in a molar ratio of 1 : 4 were dissolved in 20 ml and 160 ml methanol, respectively. Then, the two solutions were mixed and stirred for 2 hours at 40 °C. Some precipitates were formed and separated by centrifugation. Finally, the obtained precipitates were washed with methanol three times and dried under vacuum at 100 °C for 1 h to give ZIF-8.

In a defined mass ratio (1–4 wt% of ZIF-8), the synthesized ZIF-8 and [THTDP][BTI] were added into a round bottom flask. The mixture was stirred and ultrasonicated for 30 min to give the porous liquid ZIF-8/[THTDP][BTI]. As shown in Fig. S1,† the obtained porous liquid ZIF-8/[THTDP][BTI] with 2 wt% of ZIF-8 is a homogeneous milk-white viscous liquid mixture.

### 2.3 Desulfurization experiment

Model diesel was prepared by dissolving Th, BT or DBT in *n*-tetradecane. A typical desulfurization process was conducted as follows. The porous liquid and model diesel in an equal mass ratio were added into a round bottom flask and were stirred vigorously at a defined temperature for a defined time. At the end of the given time, the stirring was turned off and the mixture was kept still to afford phase separation. The oil phase was located on the top of the denser porous liquid. The sulfur content in the oil phase was determined to calculate the removal.

### 2.4 Analytical methods

The sulfur content in model diesel after desulfurization was determined using a gas chromatography/mass spectrometer (GC/MS, Agilent 7890B-5977A) equipped with an HP-5 capillary column. The carrier gas was helium. Analysis conditions are listed as follows: injector temperature: 280 °C for Th and BT and 300 °C for DBT; oven temperature: 60 °C to 280 °C at 15 °C min^−1^ for Th, 100 °C to 280 °C at 15 °C min^−1^ for BT, and 100 °C to 300 °C at 15 °C min^−1^ for DBT; injection volume: 1 μl. Each sample's sulfur content was presented as an average of three replicate (data error <5%). ZIF-8 was characterized by X-ray diffraction (XRD, Rigaku MiniFlex 600) using Cu-Kα radiation (*l* = 0.15418 nm), Fourier transform infrared spectroscopy (FTIR, Nicolet 6700, Thermo Scientific) and scanning electron microscopy (SEM, SU8010, Hitachi). The chemical structure of [THTDP][BTI] was analyzed on a nuclear magnetic resonance spectrometer (NMR, Bruker Avance III 400) using D_2_O as a solvent. Densities were measured using a U-shaped vibrating-tube densimeter (Anton Paar DMA 5000 M) under atmospheric pressure and at 308.15 K. The temperature of the measuring cell was kept constant to ±0.01 K at a defined temperature. The uncertainty of density values was less than 5 × 10^−6^ g cm^−3^.

### 2.5 Simulation details

The conformation search at the GFN2-xTB semiempirical level followed by density functional theory (DFT) calculations was used to obtain the optimal interaction of [THTDP][BTI] with ZIF-8 and BT.^36^ Zn(im)_4_ (Zn for zinc, im for imidazolate) was used to represent ZIF-8. The configurations of [THTDP][BTI], Zn(im)_4_ and BT are shown in Fig. S2.† A complex of [THTDP][BTI] and Zn(im)_4_ in a molar ratio of 1 : 1 (ZIF-8/[THTDP][BTI]) and a complex of [THTDP][BTI], Zn(im)_4_ and BT in a molar ratio of 1 : 1 : 1 (ZIF-8/[THTDP][BTI]⋯BT) were submitted to the iMTD-GC workflow for conformer search implemented in the Crest program.^37^ The total meta-dynamics simulation time was 600 ps for each system. The best conformers were optimized at the B3LYP/def2-SVP level with Grimme's D3BJ empirical dispersion correction.^38^ The single point energies were obtained at the B3LYP/def2-TZVP level. All DFT calculations were performed using the Gaussian 16 program.^39^ Independent gradient model based on Hirshfeld partition (IGMH) was performed using the Multiwfn program.^40,41^

## 3. Results and discussion

### 3.1 Characterization in relation to the porous liquid ZIF-8/[THTDP][BTI]

**Fig. 1:**
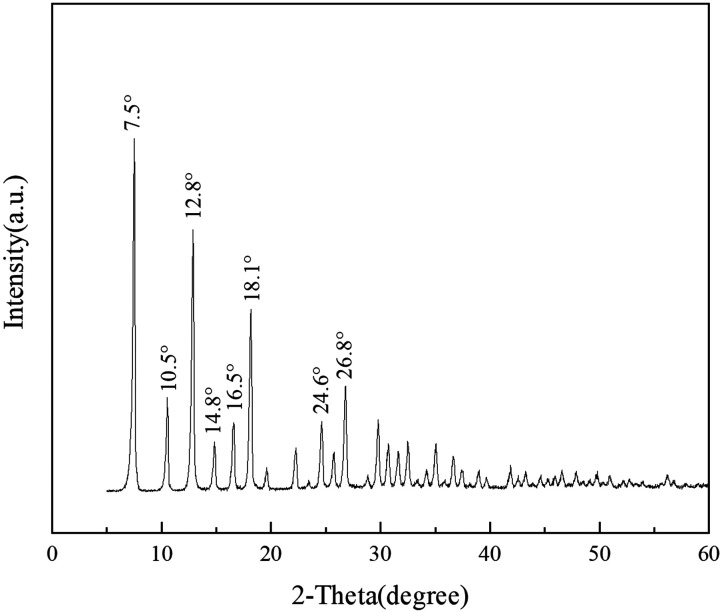
XRD pattern of pristine ZIF-8.

**Fig. 2:**
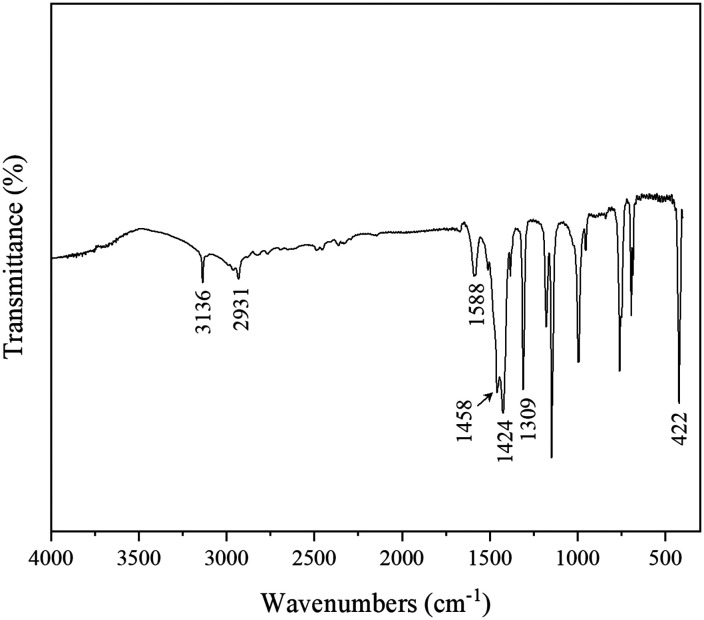
FTIR spectrum of pristine ZIF-8.

**Fig. 3:**
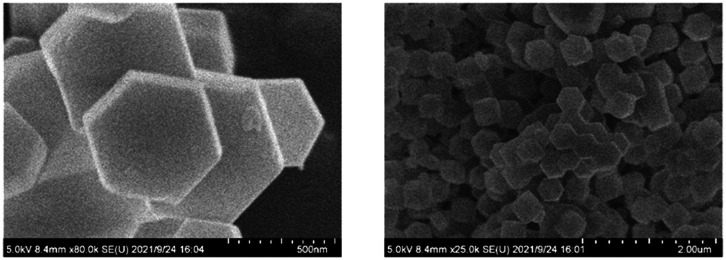
SEM images of ZIF-8.

**Fig. 4:**
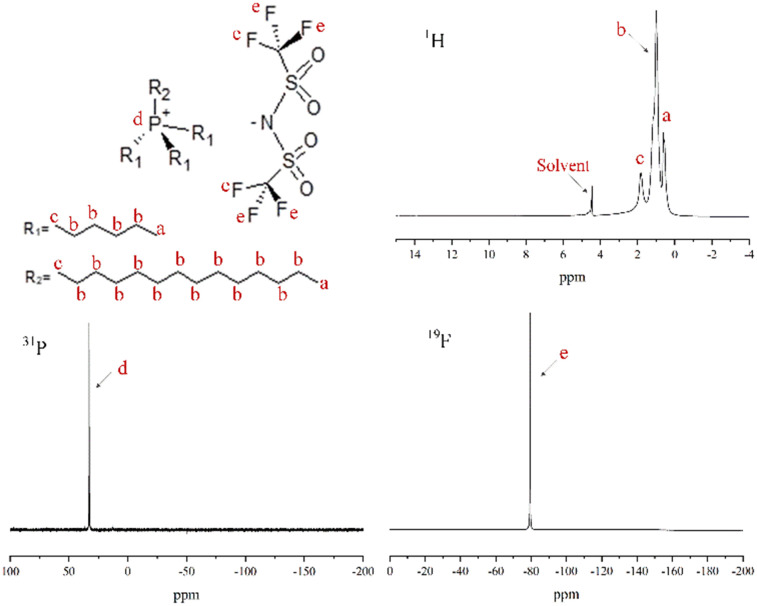
^1^H, ^31^P, and ^19^F NMR spectra of the ionic liquid [THTDP][BTI] in D_2_O.

The structure of ZIF-8 for synthesizing porous liquids was characterized by XRD, and the diffractogram in Fig. 1 shows the eight major characteristic peaks at 2*θ* of 7.5, 10.5, 12.8, 14.8, 16.5, 18.1, 24.6 and 26.8°, which was in good accordance with previous reports on ZIF-8.^42–44^ Also, the FTIR spectrum of ZIF-8 in Fig. 2 is in good agreement with a previous report.^45^ The IR bands at 3136 cm^−1^ and 2931 cm^−1^ are attributed to the stretching vibration of C–H bonds of the methyl group and the imidazole ring, respectively. The band at 1588 cm^−1^ stems from the axial deformation of C=N in the imidazole ring. The bands at 1458 cm^−1^, 1424 cm^−1^ and 1309 cm^−1^ are the characteristic stretching vibrations of the imidazole species. The band at 422 cm^−1^ is assigned to the axial deformation of Zn–N. The SEM images of ZIF-8 in Fig. 3 display a quasi-hexagonal block shape with a uniform particle size of 406 ± 21 nm, similar to the previous study.^26^ The ^1^H, ^31^P and ^19^F NMR analyses were performed to characterize the chemical structure of [THTDP][BTI]. In Fig. 4, the corresponding ^1^H, ^31^P and ^19^F NMR spectra are shown and they are in a good match with the molecular chemical structure of [THTDP][BTI].^46,47^ The proton signals located in the high field of 0–2 ppm are assigned to the alkyl protons. The presence of mono signals at 32.8 ppm and −79.3 ppm in the ^31^P and ^19^F NMR spectra, respectively, in particular confirms the high purity of [THTDP][BTI].

In order to prove that the voluminous [THTDP][BTI] molecules cannot enter the cavities of ZIF-8 and the pores of ZIF-8 in the porous liquid remain unfilled, the molecular volume of [THTDP][BTI] was calculated with Multiwfn. The volume of the cation [THTDP] is calculated to be 11.355 × 13.082 × 21.337 Å^3^ and that of the anion [BTI] is 6.707 × 6.295 × 9.493 Å^3^. Both are larger than the pore opening size of ZIF-8 (3.4 Å), proving that steric hindrance will exclude [THTDP][BTI] from the pores of ZIF-8. In addition, the densities of the pure ionic liquid [THTDP][BTI] and the porous liquid ZIF-8/[THTDP][BTI] were measured. At 308.15 K, the densities of [THTDP][BTI] and ZIF-8/[THTDP][BTI] with 2 wt% and 3 wt% contents of ZIF-8 are 1.063556 g cm^−3^, 1.063500 g cm^−3^ and 1.062096 g cm^−3^, respectively. This implies that the densities of the two porous liquids are less than that of the pure ionic liquid, apparently caused by the unfilled pores of ZIF-8 in the porous liquids.

### 3.2 Comparison of desulfurization performance

**Fig. 5:**
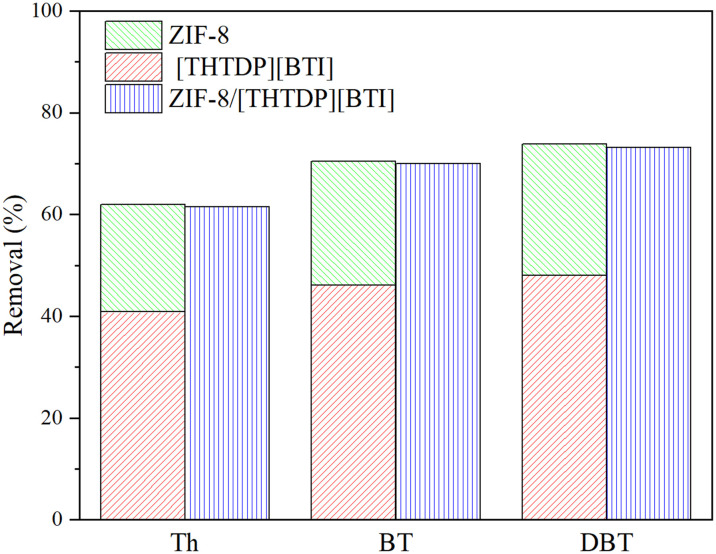
Comparison of desulfurization performance of ZIF-8, [THTDP][BTI] and ZIF-8/[THTDP][BTI] for three model organic sulfides (initial sulfur concentration = 1000 ppm, temperature = 35 °C, time = 6 h).

According to previous reports,^32–34,48^ both the phosphonium-based ionic liquids and ZIF-8 are efficient desulfurizers. Their individual desulfurization performances were compared with the porous liquid formed by mixing them, to identify the individual contribution of ZIF-8 and [THTDP][BTI] in porous liquids to the total removal. 0.02 g ZIF-8, 0.98 g [THTDP][BTI] and their mixture ZIF-8/[THTDP][BTI] with 2 wt% content of ZIF-8 were used for the desulfurization of 1 g of model diesel, respectively. As shown in Fig. 5, the removal of individual organic sulfide by the porous liquid ZIF-8/[THTDP][BTI] is almost equal to the sum of the removal by [THTDP][BTI] and by ZIF-8. For example, the removal (61.5%) of Th by ZIF-8/[THTDP][BTI] is close to the sum (61.9%) of the removal by ZIF-8 (21.0%) and by [THTDP][BTI] (40.9%). Similarly, the removal values of BT by ZIF-8, [THTDP][BTI] and ZIF-8/[THTDP][BTI] are 24.3%, 46.1% and 70.0%, respectively, and those for DBT are 25.8%, 48.1% and 73.1%, respectively. This result indicates that ZIF-8 and [THTDP][BTI] in the porous liquids individually contribute to the total removal and ZIF-8 and [THTDP][BTI] in the porous liquid maintain their original desulfurization capacities. In other words, the desulfurization effect by the porous liquid ZIF-8/[THTDP][BTI] is a joint effect of both the adsorptive desulfurization by ZIF-8 and the extraction desulfurization by [THTDP][BTI].

It is worth noting that the removal of the three model organic sulfides by ZIF-8/[THTDP][BTI] follows the order of DBT (73.1%) > BT (70.0%) > Th (61.5%), which is in good agreement with the order of their molecular weights (DBT = 184.3 g mol^−1^, BT = 134.2 g mol^−1^, Th = 84.1 g mol^−1^). This implies that the physical interaction is probably the major driving force for the separation of organic sulfides. Therefore, it can be inferred that van der Waals forces are the main interactions between ZIF-8/[THTDP][BTI] and organic sulfides. The inference will be further investigated by theoretical calculations later.

### 3.3 Effect of experimental factors on the removal of organic sulfides

**Fig. 6:**
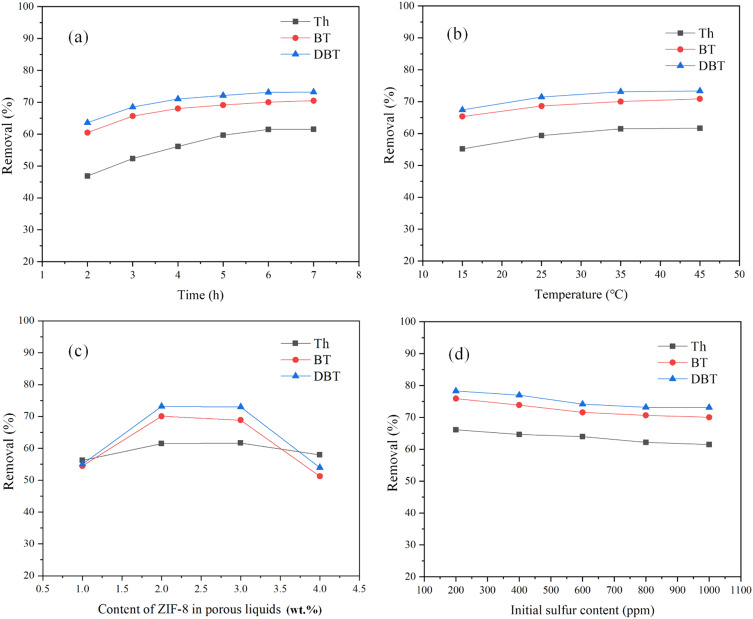
Effects of experimental factors on the removal of organic sulfides. (a) Time (temperature = 35 °C, content of ZIF-8 = 2 wt%, initial sulfur content = 1000 ppm), (b) temperature (time = 6 h, content of ZIF-8 = 2 wt%, initial sulfur content = 1000 ppm), (c) the content of ZIF-8 in porous liquids (temperature = 35 °C, time = 6 h, initial sulfur content = 1000 ppm), and (d) initial sulfur content (temperature = 35 °C, time = 6 h, content of ZIF-8 = 2 wt%).

In Fig. 6, the effects of experimental factors such as time, temperature, the content of ZIF-8 and initial sulfur content on the removal of organic sulfides are presented. As shown from the trends in Fig. 6a, the removal of all three organic sulfides increased with time within the first 6 h, after which the removal remained practically unchanged. The removal of the three organic sulfides follows the order of DBT > BT > Th at any testing time. The removal of all three organic sulfides was observed to increase slightly with the rise of reaction temperature, as can be seen from Fig. 6b. This indicates that ZIF-8/[THTDP][BTI] remained stable for the removal of organic sulfides over a wide temperature range, which could be related to the stability of [THTDP][BTI]. Next, the content of ZIF-8 in the porous liquid was studied, and as shown in Fig. 6c, it has a significant impact on the removal. At a low content of ZIF-8 (<3 wt%), the removal of all three organic sulfides increases with the rising content of ZIF-8. This tendency is expected as long as the organic sulfides are preferably partitioned on ZIF-8. However, at a content of ZIF-8 >3 wt%, the removal decreased with an increasing content of ZIF-8, which could potentially be related to mass transfer inefficiencies related to the aggregation of the ZIF-8 particles in the porous liquid. Regarding the initial sulfur content, it has a limited impact on the removal, as shown in Fig. 6d. The removal of three organic sulfides increases only slightly with the change of the initial sulfur content. Therefore, the porous liquid ZIF-8/[THTDP][BTI] can be used for the desulfurization of diesel with a wide range of sulfur content.

### 3.4 Desulfurization mechanism

To pinpoint the desulfurization mechanism, ZIF-8/[THTDP][BTI] and ZIF-8/[THTDP][BTI]⋯BT (defined as the porous liquid carrying the captured organic sulfide BT) were optimized by conformation search and DFT calculations. The optimized configurations of ZIF-8/[THTDP][BTI] and ZIF-8/[THTDP][BTI]⋯BT are displayed in Fig. S3 and S4.† IGMH analysis was carried out to reveal the interactions between ZIF-8/[THTDP][BTI] and BT. ZIF-8/[THTDP][BTI] and BT were treated as two fragments. The isosurfaces of *δg*^inter^ are used for the visualization of interaction regions. *δg*^inter^ can be defined by the following eqn (1)–(3), where *δg*^inter^ is the inter-fragment interaction, *g*^inter^ is the magnitude of superposition of density gradient of all fragments, *g*^IGM,inter^ is the sum of magnitude of density gradient of all fragments, *r* denotes the Cartesian coordinate vector, *A* loops over all fragments, *ρ*^free^_*i*_ stands for the spherically averaged density of atom *i* in its free state, and *i* loops all atoms in the corresponding fragment.

1 *δg*^inter^(*r*) = *g*^IGM,inter^(*r*) − *g*^inter^(*r*)

2
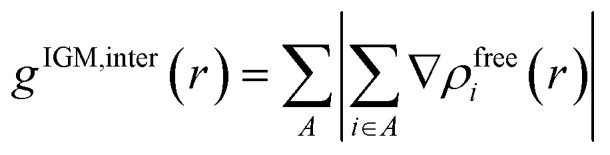
3
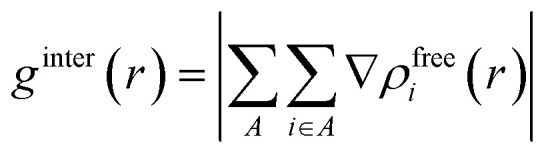
The function of sign(*λ*_2_)*ρ* can be utilized to distinguish the type and intensity of the interaction and can be mapped on the isosurfaces with different colors to visually characterize the nature of the interactions, where *ρ* is the electron density and *λ*_2_ is the second largest eigenvalue of the Hessian matrix.

**Fig. 7:**
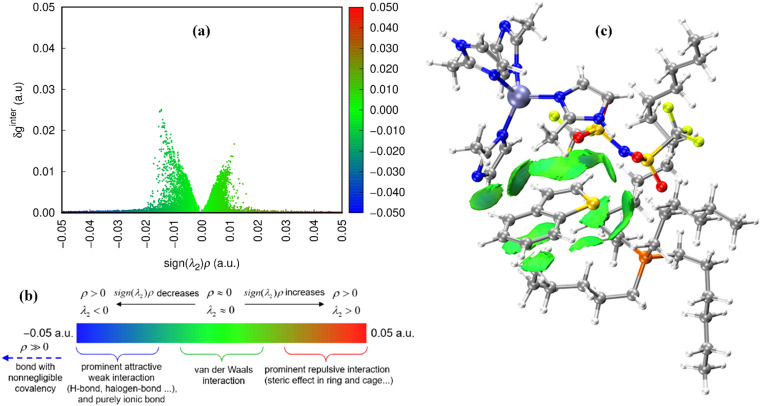
(a) The scatter plot of *δg vs.* sign(*λ*_2_)*ρ*. (b) The coloring method of mapped function sign(*λ*_2_)*ρ* in IGMH maps. (c) Sign(*λ*_2_)*ρ* colored isosurfaces of *δg*^inter^ = 0.005 a.u. of ZIF-8/[THTDP][BTI]⋯BT corresponding to IGMH analyses.

**Fig. 8:**
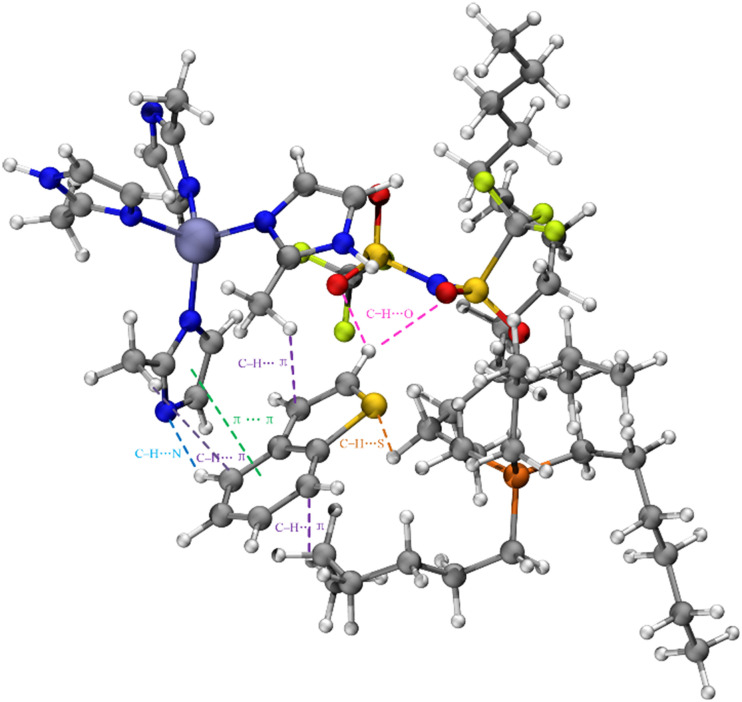
The decomposition of the van der Waals interactions between ZIF-8/[THTDP][BTI] and BT.

Fig. 7b exhibits the coloring method of mapped function sign(*λ*_2_)*ρ* in IGMH maps. The blue region represents prominent attractive interactions such as H-bonding, the green region indicates van der Waals interactions, and the red region represents prominent repulsive interactions such as steric effects. Fig. 7a visualizes the scatter plot of *δg*^inter^*vs.* sign(*λ*_2_)*ρ*. The values of sign(*λ*_2_)*ρ* are concentrated near zero, which indicates that the van der Waals interactions are the main interactions that are active between ZIF-8/[THTDP][BTI] and BT. Furthermore, Fig. 7c illustrates the sign(*λ*_2_)*ρ* colored isosurfaces of *δg*^inter^ = 0.005 a.u. of ZIF-8/[THTDP][BTI]⋯BT corresponding to IGMH analyses, where the types and regions of the interactions between ZIF-8/[THTDP][BTI] and BT are further clearly revealed. As shown in Fig. 7c, many large green slides exist between ZIF-8/[THTDP][BTI] and BT, which also indicates that the van der Waals interaction is the main interaction between ZIF-8/[THTDP][BTI] and BT. Therefore, this result confirms the aforementioned inference based on the experimental results. In addition, according to previous studies,^48–50^ the van der Waals interactions between ZIF-8/[THTDP][BTI] and BT were further decomposed. As presented in Fig. 8, the van der Waals interactions between ZIF-8/[THTDP][BTI] and BT include the interactions of C–H⋯O between BT and [BTI], the interactions of C–H⋯π, π⋯π, and C–H⋯N between BT and Zn(im)_4_, and the interactions of C–H⋯π and C–H⋯S between BT and [THTDP].

### 3.5 Multiple desulfurization

**Fig. 9:**
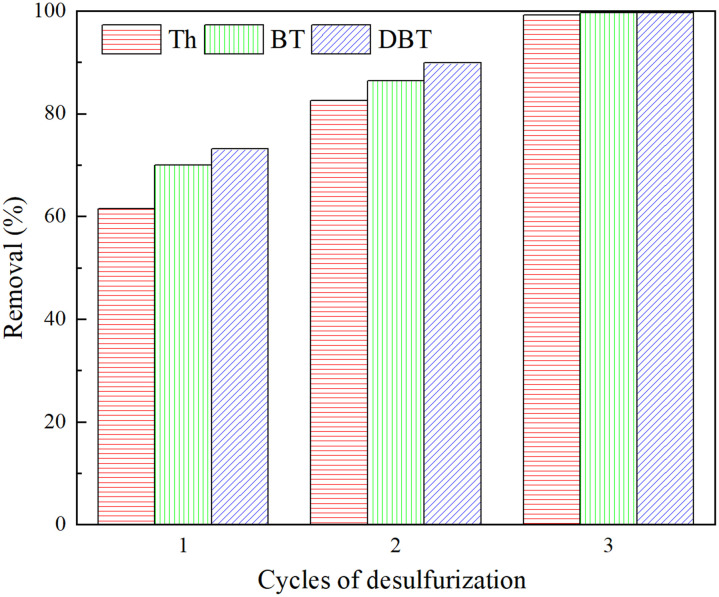
Triple cycle desulfurization of model diesel with the porous liquid ZIF-8/[THTDP][BTI] (temperature = 35 °C, time = 6 h, content of ZIF-8 = 2 wt%, initial sulfur content = 1000 ppm).

Multiple cycles of desulfurization would be needed to fulfill deep desulfurization for satisfying the conditions of desulfurization set by regulatory bodies. Here, model diesel was desulfurized with the freshly prepared porous liquids for three consecutive cycles. As shown in Fig. 9, the desulfurization degree increases cycle by cycle. After three cycles of desulfurization, the removal reaches close to 100% for all three organic sulfides. That is, deep desulfurization can be satisfactorily fulfilled by our porous liquid through only triple desulfurization cycles.

### 3.6 Recycling and regeneration of ZIF-8/[THTDP][BTI]

**Fig. 10:**
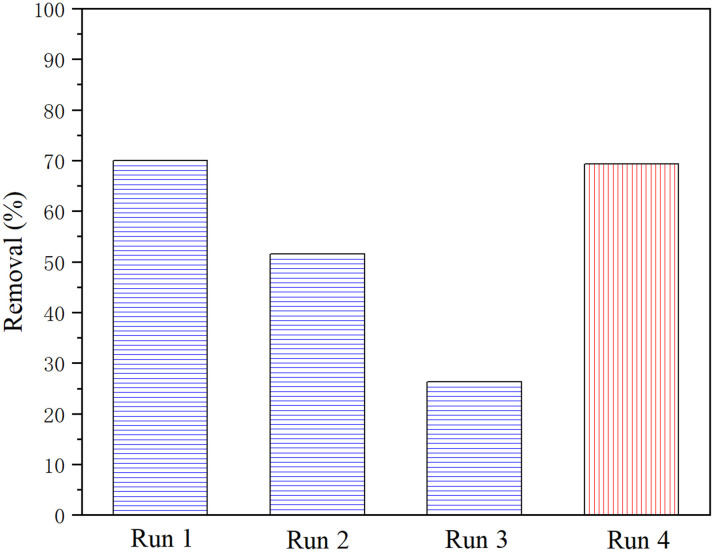
Recycling and regeneration of ZIF-8/[THTDP][BTI] in 4 cycle tests (temperature = 35 °C, time = 6 h, content of ZIF-8 = 2 wt%, initial sulfur content = 1000 ppm).

**Fig. 11:**
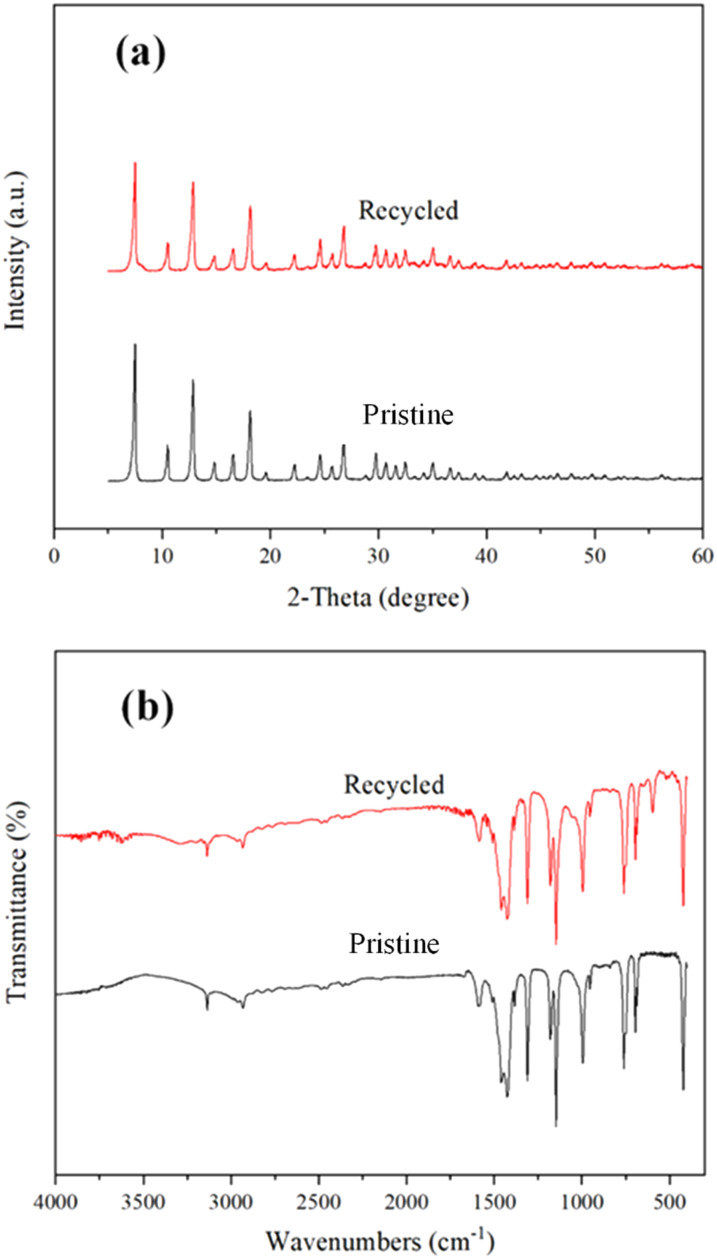
(a) XRD patterns and (b) FTIR spectra of pristine and recycled ZIF-8.

The recycling and regeneration of ZIF-8/[THTDP][BTI] were investigated by taking BT as an example. 4 g of ZIF-8/[THTDP][BTI] was recycled for the desulfurization of model diesel containing BT for four runs. Briefly, the porous liquid was used for three consecutive runs without any treatment between each run. After the third run, the porous liquid was stripped with pure *n*-tetradecane and then used for a fourth run. As indicated in Fig. 10, the removal decreases from 70.0% to 26.3% after three consecutive runs. However, the removal returns to 69.4% in the fourth run. This means that the regeneration of ZIF-8/[THTDP][BTI] is possible and can be fulfilled through stripping with pure *n*-tetradecane. In addition, the ZIF-8 separated from the recovered ZIF-8/[THTDP][BTI] after the fourth run was characterized by XRD and FTIR. As compared in Fig. 11, the XRD pattern and FTIR spectrum of the recycled ZIF-8 are practically identical to those of the pristine ZIF-8, which shows that ZIF-8 remains chemically unchanged during desulfurization and regeneration. Perfect regeneration performance is a crucial feature to support ZIF-8/[THTDP][BTI] as a potential desulfurization agent for industrial application.

## 4. Conclusions

A type-III porous liquid was successfully prepared by dispersing ZIF-8 in ionic liquid [THTDP][BTI] and used for the desulfurization of model diesel. The sterically hindered solvent [THTDP][BTI] is dimensionally excluded from the cavities of ZIF-8, and thus the pores of ZIF-8 in porous liquids remain accessible to organic sulfides. The desulfurization effect by the porous liquid ZIF-8/[THTDP][BTI] is a joint effect of both the adsorptive desulfurization by ZIF-8 and the extraction desulfurization by [THTDP][BTI]. At 35 °C and an initial sulfur content of 1000 ppm, the removal values of DBT, BT and Th are 73.1%, 70.0% and 61.5%, respectively, within 6 h at a content of ZIF-8 of 2 wt%. The removal of all three organic sulfides reaches nearly 100% after three cycles of desulfurization. The regeneration of ZIF-8/[THTDP][BTI] could be uncomplicatedly fulfilled by stripping with pure *n*-tetradecane, and the chemical structure of ZIF-8 remained intact after the desulfurization and regeneration operations. Both the experimental results and theoretical calculation indicate that van der Waals interactions were the main interactions that are active between ZIF-8/[THTDP][BTI] and BT. Our discovery here demonstrates that the porous liquid ZIF-8/[THTDP][BTI] carries great potential for industrial desulfurization application.

## Author contributions

Chenhua Shu: conceptualization, investigation, formal analysis, resources, data curation, writing-original draft, funding acquisition, and project administration. Min Zhao: investigation and funding acquisition. Hua Cheng: investigation, formal analysis, and funding acquisition. Yajie Deng: investigation, data curation, and funding acquisition. Pierre Stiernet: discussion and writing-review & editing. Niklas Hedin: discussion and writing-review & editing. Jiayin Yuan: conceptualization, discussion, writing-review & editing, and supervision.

## Conflicts of interest

There are no conflicts to declare.

## Supplementary Material

RE-008-D3RE00364G-s001

## References

[cit1] Haruna A., Merican Z. M. A., Musa S. G., Abubakar S. (2022). Fuel.

[cit2] Ullah L., Zhao G. Y., Hedin N., Ding X. L., Zhang S. J., Yao X. Q., Nie Y., Zhang Y. Q. (2019). Chem. Eng. J..

[cit3] Boshagh F., Rahmani M., Zhu W. S. (2022). Energy Fuels.

[cit4] Lee K. X., Valla J. A. (2019). React. Chem. Eng..

[cit5] Abro R., Kiran N., Ahmed S., Muhammad A., Jatoi A. S., Mazari S. A., Salma U., Plechkova N. V. (2022). J. Environ. Chem. Eng..

[cit6] Liu X. J., Li J. W., Guo Y. W., Wu J., Hu B. (2022). React. Chem. Eng..

[cit7] Zaidi Z., Sorokhaibam L. G. (2022). Energy Fuels.

[cit8] Omar R. A., Verma N. (2022). Ind. Eng. Chem. Res..

[cit9] Ganiyu S. A., Lateef S. A. (2021). Fuel.

[cit10] Hao L. D., Hurlock M. J., Ding G. D., Zhang Q. (2020). Top. Curr. Chem..

[cit11] Crandall B. S., Zhang J. Y., Stavila V., Allendorf M. D., Li Z. L. (2019). Ind. Eng. Chem. Res..

[cit12] He S. F., Chen L. H., Cui J., Yuan B., Wang H. L., Wang F., Yu Y., Lee Y., Li T. (2019). J. Am. Chem. Soc..

[cit13] O'Reilly N., Giri N., James S. L. (2007). Chem. – Eur. J..

[cit14] Fulvio P. F., Dai S. (2020). Chem.

[cit15] Egleston B. D., Mroz A., Jelfs K. E., Greenaway R. L. (2022). Chem. Sci..

[cit16] Li Y. (2020). ChemistrySelect.

[cit17] Yin J., Zhang J. R., Fu W. D., Ran H. S., Zhang Y., Zhang M., Jiang W., Li H. P., Zhu W. S., Li H. M. (2022). Sep. Purif. Technol..

[cit18] Zhang J. H., Wei M. J., Lu Y. L., Wei Z. W., Wang H. P., Pan M. (2020). ACS Appl. Energy Mater..

[cit19] Wang D. C., Xin Y. Y., Yao D. D., Li X. Q., Ning H. L., Zhang H. M., Wang Y. D., Ju X. Q., He Z. J., Yang Z. Y., Fan W. D., Li P. P., Zheng Y. P. (2022). Adv. Funct. Mater..

[cit20] Shan W., Fulyio P. F., Kong L. Y., Schott J. A., Do-Thanh C. L., Tian T., Hu X. X., Mahurin S. M., Xing H. B., Dai S. (2018). ACS Appl. Mater. Interfaces.

[cit21] Jie K. C., Onishi N., Schott J. A., Popovs I., Jiang D. E., Mahurin S., Dai S. (2020). Angew. Chem., Int. Ed..

[cit22] Lai B. B., Cahir J., Tsang M. Y., Jacquemin J., Rooney D., Murrer B., James S. L. (2021). ACS Appl. Mater. Interfaces.

[cit23] Zhang Z. X., Yang B. L., Zhang B. J., Cui M. F., Tang J. H., Qiao X. (2022). Nat. Commun..

[cit24] Li X., Wang D., He Z., Su F., Zhang N., Xin Y., Wang H., Tian X., Zheng Y., Yao D., Li M. (2021). Chem. Eng. J..

[cit25] Giri N., Del Pópolo M. G., Melaugh G., Greenaway R. L., Rätzke K., Koschine T., Pison L., Costa Gomes M. F., Cooper A. I., James S. L. (2015). Nature.

[cit26] Wu J. M., Wu X. M., Zhao P. P., Wang Z. H., Zhang L. Z., Xu D. M., Gao J. (2021). Fuel.

[cit27] Yu G. J., Jin D. Y., Zhang F., Tian S. C., Zhou Z. Y., Ren Z. Q. (2023). Chem. Eng. J..

[cit28] Zhang J. R., Yin J., Fu W. D., Ran H. S., Jiang W., Li H. P., Zhu W. S., Li H. M., Zhang M. (2023). Fuel Process. Technol..

[cit29] Wang B. S., Qin L., Mu T. C., Xue Z. M., Gao G. H. (2017). Chem. Rev..

[cit30] Baumann M. D., Daugulis A. J., Jessop P. G. (2005). Appl. Microbiol. Biotechnol..

[cit31] Moghadam F. R., Azizian S., Kianpour E., Yarie M., Bayat M., Zolfigol M. A. (2017). Chem. Eng. J..

[cit32] Gomes M. C., Pison L., Cervinka C., Padua A. (2018). Angew. Chem., Int. Ed..

[cit33] Ahmed O. U., Mjalli F. S., Gujarathi A. M., Al-Wahaibi T., Al- Wahaibi Y., Ai-Nashef I. M. (2015). Fluid Phase Equilib..

[cit34] Moghadam F. R., Azizian S., Bayat M., Yarie M., Kianpour E., Zolfigol M. A. (2017). Fuel.

[cit35] Dharaskar S., Sillanpaa M., Tadi K. K. (2018). Environ. Sci. Pollut. Res..

[cit36] Bannwarth C., Ehlert S., Grimme S. (2019). J. Chem. Theory Comput..

[cit37] Pracht P., Bohle F., Grimme S. (2020). Phys. Chem. Chem. Phys..

[cit38] Grimme S., Ehrlich S., Goerigk L. (2011). J. Comput. Chem..

[cit39] Frisch M. J. , Trucks G. W., Schlegel H. B., Scuseria G. E., Robb M. A., Cheeseman J. R., Scalmani G., Barone V., Petersson G. A., Nakatsuji H., Li X., Caricato M., Marenich A. V., Bloino J., Janesko B. G., Gomperts R., Mennucci B., Hratchian H. P., Ortiz J. V., Izmaylov A. F., Sonnenberg J. L., Williams-Young D., Ding F., Lipparini F., Egidi F., Goings J., Peng B., Petrone A., Henderson T., Ranasinghe D., Zakrzewski V. G., Gao J., Rega N., Zheng G., Liang W., Hada M., Ehara M., Toyota K., Fukuda R., Hasegawa J., Ishida M., Nakajima T., Honda Y., Kitao O., Nakai H., Vreven T., Throssell K., Montgomery Jr J. A.., Peralta J. E., Ogliaro F., Bearpark M. J., Heyd J. J., Brothers E. N., Kudin K. N., Staroverov V. N., Keith T. A., Kobayashi R., Normand J., Raghavachari K., Rendell A. P., Burant J. C., Iyengar S. S., Tomasi J., Cossi M., Millam J. M., Klene M., Adamo C., Cammi R., Ochterski J. W., Martin R. L., Morokuma K., Farkas O., Foresman J. B. and Fox D. J., Gaussian 16 Revision C.01, 2016

[cit40] Lu T., Chen Q. (2022). J. Comput. Chem..

[cit41] Lu T., Chen F. (2012). J. Comput. Chem..

[cit42] Cheng T. J., Zhang Y. H., Cui F. J., Jiang G., Liu P. Z., Guo J., Cui K., Chen C., Li H. D. (2022). Chem. Phys. Lett..

[cit43] Zhou L., Li N., Owens G., Chen Z. (2019). Chem. Eng. J..

[cit44] Kaur H., Mohanta G. C., Gupta V., Kukkar D., Tyagi S. (2017). J. Drug Delivery Sci. Technol..

[cit45] Han X., Hu T., Wang Y., Chen H., Wang Y., Yao R., Ma X., Li J., Li X. (2018). Sep. Purif. Technol..

[cit46] Cholico-Gonzalez D., Avila-Rodriguez M., Cote G., Chagnes A. (2013). J. Mol. Liq..

[cit47] Li N., Zhang S. H., Zheng L. Q., Inoue T. (2009). Langmuir.

[cit48] Khan N. A., Bhadra B. N., Jhung S. H. (2018). Chem. Eng. J..

[cit49] Lu P., Yin M. L., Chen J., Wang Q. L., Ye C. S., Qiu T. (2022). Chem. Eng. J..

[cit50] Zhao H., Baker G. A., Wagle D. V., Ravula S., Zhang Q. (2016). ACS Sustainable Chem. Eng..

