# Supplementary material for: Desulfurization of diesel via joint adsorption and extraction using a porous liquid derived from ZIF-8 and a phosphonium-type ionic liquid
Source: React Chem Eng. 2023 Aug 4;8(12):3124–32. doi: 10.1039/d3re00364g (PMC10660146; doi:10.1039/d3re00364g)
Supplement: RE-008-D3RE00364G-s001 [file RE-008-D3RE00364G-s001.pdf]

Electronic Supplementary Material (ESI) for Reaction Chemistry & Engineering.
This journal is © The Royal Society of Chemistry 2023

Electronic supplementary information for

Desulfurization of diesel *via* joint adsorption and extraction using a porous liquid derived from ZIF-8 and a phosphonium-type ionic liquid

Chenhua Shu,*[ab] Min Zhao,[a] Hua Cheng,[a] Yajie Deng,[a] Pierre Stiernet,[b] Niklas Hedin[b] and Jiayin Yuan*[b]

[a]*School of Chemistry and Environmental Science, Shangrao Normal University, Shangrao 334001, China*

[b]*Department of Materials and Environmental Chemistry, Stockholm University, Stockholm, 10691 Sweden*

* Corresponding author.

E-mail address: chenhua.shu@mmk.su.se; jiayin.yuan@mmk.su.se

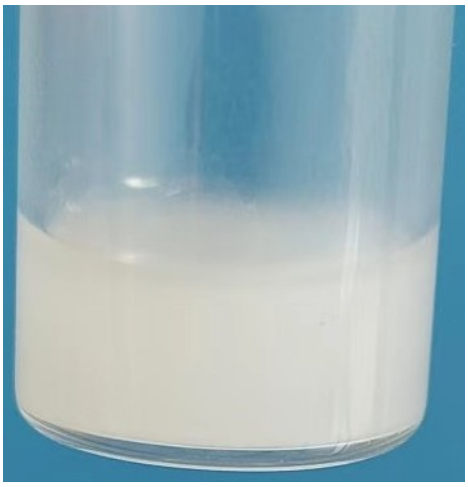

Fig. S1. The photograph of the porous liquid with 2 wt.% content of ZIF-8 in [THTDP][BTI].

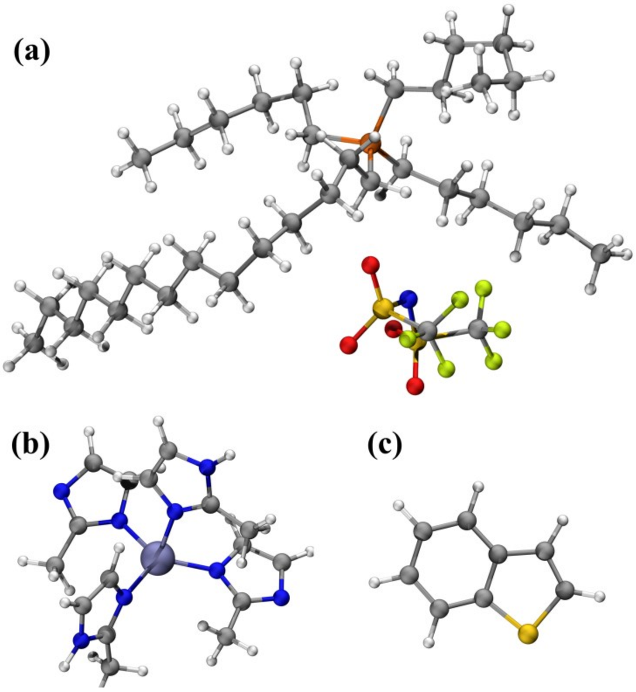

Fig. S2. Configurations of (a) [THTDP][BTI], (b) $Zn(im)_4$ and (c) BT. Carbon atoms are displayed in grey, hydrogen in grayish white, phosphorus in orange, nitrogen in blue, sulfur in yellow, oxygen in red, fluorine in green, and zinc in grey blue.

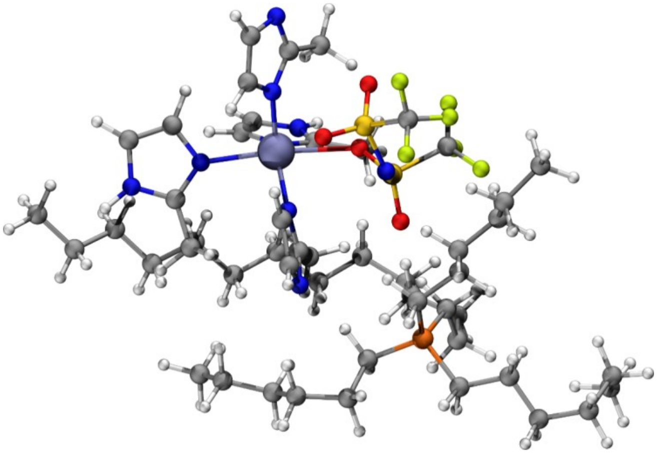

Fig. S3. Optimized configurations of ZIF-8/[THTDP][BTI].

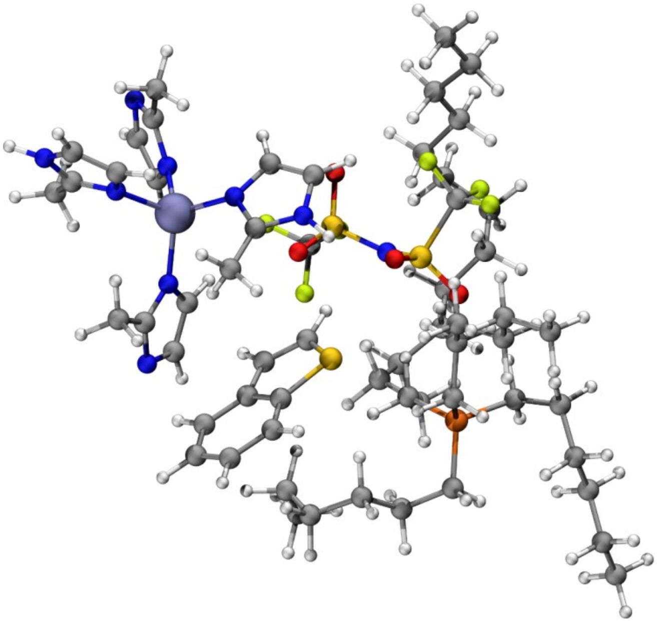

Fig. S4. Optimized configurations of ZIF-8/[THTDP][BTI]···BT.
